# Scleredema Diabeticorum Mimicking Systemic Sclerosis in a 75‐Year‐Old Female: A Case Report

**DOI:** 10.1155/crdm/1671656

**Published:** 2026-11-11

**Authors:** Muhammad Waleed Imran, Sanwal Sardar Nawaz, Hina Abbasi, Farah Khurshid, Waqar Khattak, Tahir Iqbal

**Affiliations:** ^1^ Shifa College of Medicine Shifa Tameer-e-Millat University Islamabad Pakistan stmu.edu.pk; ^2^ Department of Internal Medicine Shifa Tameer-e-Millat University Islamabad Pakistan stmu.edu.pk; ^3^ Department of Nephrology Shifa Tameer-e-Millat University Islamabad Pakistan stmu.edu.pk

## Abstract

Scleredema diabeticorum is a rare sclerosing cutaneous disorder characterized by diffuse, nonpitting, woody skin induration, most commonly occurring in patients with long‐standing diabetes mellitus. Its clinical resemblance to systemic sclerosis (SSc) may result in diagnostic confusion, particularly in atypical presentations. We report a 75‐year‐old woman with a 20‐year history of insulin‐dependent Type 2 diabetes mellitus who developed progressive, generalized skin thickening. The disease initially involved the lower extremities and subsequently progressed proximally to the trunk, upper limbs, and face, with prominent facial and periorbital involvement and sparing of the hands and feet. She was initially suspected to have SSc because of diffuse skin induration and positive antinuclear antibodies and was treated with corticosteroids. However, the absence of characteristic distal scleroderma features, negative SSc‐specific antibody testing, and histopathological findings of marked dermal thickening with broad collagen bundles and mucin deposition supported a diagnosis of scleredema diabeticorum. Management was complicated by diabetic nephropathy and concurrent cardiac disease, requiring individualized immunomodulatory therapy, close monitoring, and optimization of glycemic control. UVA1 phototherapy was attempted but discontinued because of immediate clinical intolerance. The patient subsequently received systemic immunomodulatory treatment with clinical improvement during follow‐up. This case highlights the importance of distinguishing scleredema diabeticorum from SSc, particularly when atypical clinical features and positive nonspecific autoantibodies may lead to diagnostic uncertainty. Careful clinical assessment, appropriate serological evaluation, and histopathology are essential for accurate diagnosis and management.

## 1. Introduction

Scleredema is a sclerosing cutaneous disorder characterized by excessive collagen deposition in the dermis, resulting in diffuse, nonpitting, woody induration of the skin. It most commonly affects middle‐aged men, typically around the age of 40 [1].

Scleredema is traditionally classified into three clinical types according to its associated conditions. Type 1, comprising approximately 55% of cases, is generally associated with a preceding febrile illness and predominantly affects children. Type 2 accounts for around 25% of cases and is most commonly associated with paraproteinemia, whereas Type 3, or scleredema diabeticorum, represents approximately 20% of cases and is strongly associated with diabetes mellitus [2]. In diabetes‐associated disease, chronic metabolic alterations are thought to promote fibroblast activation, increased collagen and mucin synthesis, and impaired collagen degradation, contributing to progressive dermal thickening and induration [3].

Scleredema diabeticorum typically involves the posterior neck, upper back, and shoulders and may extend to the face and upper extremities. The affected skin is often firm and may exhibit a characteristic “peau d’orange” texture. Histopathologically, scleredema is characterized by thickening of the dermis with broad collagen bundles and interstitial mucin deposition, while the epidermis appears thinned [4, 5].

The cutaneous manifestations of scleredema closely resemble those of systemic sclerosis (SSc) and may therefore lead to misdiagnosis, with definitive distinction often requiring skin biopsy. The distribution of skin involvement can aid differentiation: scleredema typically spares the distal extremities such as the hands and feet, whereas scleroderma frequently involves these areas. Treatment of scleredema is challenging, as no standardized regimen exists. Reported therapies include tight glycemic control, phototherapy (such as UVA1 or PUVA), systemic corticosteroids, and immunosuppressants like methotrexate or cyclosporine. Responses vary widely among patients, and outcomes are often unpredictable, necessitating individualized, multidisciplinary management based on disease severity and systemic involvement [1].

We describe a 75‐year‐old insulin‐dependent diabetic woman who developed progressive skin induration and edema, initially thought to have SSc but ultimately diagnosed with scleredema diabeticorum by biopsy.

## 2. Case Report

A 75‐year‐old woman with a 20‐year history of Type 2 diabetes mellitus requiring insulin therapy (recent HbA1c level of 7.8%) and hypertension presented to the emergency department with complaints of drowsiness, nausea, vomiting, fever (38.9°C), increased urinary frequency, and profuse sweating. Initial laboratory evaluation revealed significant hyperglycemia (324 mg/dL), hyponatremia (serum sodium level of 126 mmol/L), and elevated cardiac enzymes consistent with a non‐ST elevation myocardial infarction (NSTEMI). Echocardiography demonstrated left ventricular dilatation with a reduced ejection fraction of 30%. She was managed supportively with electrolyte correction, anticoagulation, and standard heart failure therapy.

On examination, diffuse skin induration was noted involving the face, trunk, and upper and lower limbs. The affected areas had a bound‐down, mildly erythematous, waxy appearance (Figure 1). Notably, the hands and feet were spared, with no evidence of digital ulcers or sclerodactyly.

**FIGURE 1:**
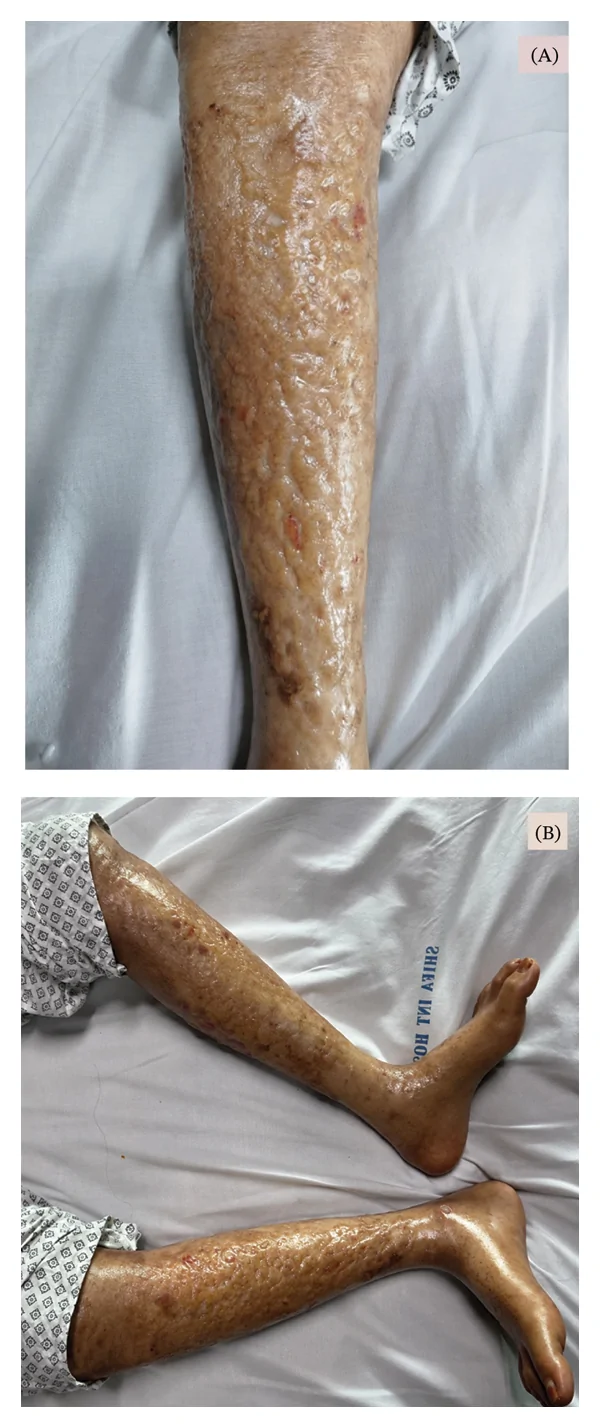
(A) High‐magnification close‐up view of the lower leg demonstrating marked dermal induration, overt surface cobblestoning, and mild persistent erythema. (B) Bilateral lower‐extremity view showing extensive skin thickening and tethering extending from the shins to the ankles with evident sparing of feet.

The patient’s history was notable for a cerebrovascular accident 1 year earlier, resulting in transient diplopia, which resolved spontaneously. Six months before the current presentation, she developed generalized body swelling, including periorbital puffiness and bilateral lower limb edema. The evaluation at the time revealed hypoalbuminemia and elevated serum creatinine, suggestive of diabetic nephropathy, for which she was prescribed furosemide. Although the edema improved, new areas of skin induration developed on her lower extremities, gradually extending upward to involve the infraumbilical region and subsequently the upper limbs and face.

The skin changes were associated with pruritus, scaling, and increasing stiffness, eventually leading to significant restriction of joint mobility. During this period, areas of yellowish discharge, erosions, and crusting developed and were attributed to secondary bacterial infection, which was managed with oral antibiotics and topical agents including polymyxin B sulfate combined with betamethasone valerate. A rheumatologist noted ANA positivity and, suspecting SSc, initiated deflazacort 30 mg daily, with a planned reduction of 6 mg per week. The patient was still on corticosteroids at the time of her emergency presentation.

Following stabilization of her acute cardiovascular and metabolic status, a more detailed evaluation of her cutaneous findings was undertaken. Autoimmune testing confirmed positive antinuclear antibody (ANA) and antismooth muscle antibody. Additional autoimmune serological testing, including anti‐dsDNA, antineutrophil cytoplasmic antibody (ANCA), and the extractable nuclear antigen (ENA) panel, was negative (Table 1). To further evaluate for SSc, an extended SSc line immunoassay (IL‐30 profile) was performed (Table 2). The panel did not detect the major SSc‐associated autoantibodies. Notably, the patient was receiving low‐dose oral deflazacort at the time of serological sampling.

**TABLE 1:** Autoantibody and complement profile of the patient.

Test	Result	Reference range/interpretation
Anti‐Sm antibodies	0.1	< 5 Negative; 5–10 Equivocal; > 10 Positive
Anti‐RNP antibodies	0.1	< 5 Negative; 5–10 Equivocal; > 10 Positive
Anti‐Sm/RNP antibodies	0.1	< 5 Negative; 5–10 Equivocal; > 10 Positive
Anti‐SSA (Ro 60 kD) antibodies	0.1	< 5 Negative; 5–10 Equivocal; > 10 Positive
Anti‐SSB (La) antibodies	0.1	< 5 Negative; 5–10 Equivocal; > 10 Positive
Anti‐Jo‐1 antibodies	0.1	< 5 Negative; 5–10 Equivocal; > 10 Positive
Anti‐Scl‐70 antibodies	0.1	< 5 Negative; 5–10 Equivocal; > 10 Positive
Anti‐PM‐Scl 100 antibodies	0.1	< 5 Negative; 5–10 Equivocal; > 10 Positive
Anti‐PM‐Scl antibodies	0.1	< 5 Negative; 5–10 Equivocal; > 10 Positive
Anti‐Ku antibodies	0.1	< 5 Negative; 5–10 Equivocal; > 10 Positive
Anti‐CENP‐A/B antibodies	0.1	< 5 Negative; 5–10 Equivocal; > 10 Positive
Anti‐PCNA antibodies	0.1	< 5 Negative; 5–10 Equivocal; > 10 Positive
Anti‐Mi‐2 antibodies	0.1	< 5 Negative; 5–10 Equivocal; > 10 Positive
ANA by IFA	1:100, 1+ fine‐speckled pattern (AC‐04)	
AMA (Antimitochondrial Antibodies)	Negative	
ASMA (Antismooth Muscle Antibodies)	Positive	
GPC (Gastric Parietal Cell Antibodies)	Negative	
LKM (Liver‐Kidney Microsomal Antibodies)	Negative	
Anti‐dsDNA Antibodies	0.1	< 5 Negative; 5–10 Equivocal; > 10 Positive
Anti‐Histone Antibodies	0.1	< 5 Negative; 5–10 Equivocal; > 10 Positive
Anti‐Nucleosome Antibodies	0.1	< 5 Negative; 5–10 Equivocal; > 10 Positive
c‐ANCA (PR3 antibody)	0.1	Negative: < 5 AU; Equivocal: 5–10 AU; Positive: > 10 AU
p‐ANCA (MPO antibody)	0.1	Negative: < 5 AU; Equivocal: 5–10 AU; Positive: > 10 AU
C4 Complement Level	0.32	Ref: 0.10–0.40 g/L
C3 Complement Level	1.25	Ref: 0.90–1.80 g/L

*Note:* Antinuclear antibody (ANA) screening was performed by indirect immunofluorescence assay (IFA) on HEp‐2 cell substrate slides at a 1:100 dilution using an automated IFA processor. Specific extractable nuclear antigen (ENA) were assessed using an automated immunodot assay on the BlueDiver Instrument (D‐tek/Alphadia, Mons, Belgium), with results evaluated by digital densitometric scanning software (Dr DOT/BlueScan). Values reported as 0.1 AU represent results below the assay’s lower limit of reportability rather than discrete measured values. ANA, antinuclear antibody; ANCA, antineutrophil cytoplasmic antibodies; RNP, ribonucleoprotein; SSA, Sjögren syndrome‐related antigen A; SSB, Sjögren syndrome‐related antigen B; PM‐Scl, polymyositis/scleroderma; CENP, centromere protein.

Abbreviations: AU, arbitrary units; IFA, indirect immunofluorescence assay; PCNA, proliferating cell nuclear antigen.

**TABLE 2:** IL‐30 autoimmune profile for systemic sclerosis.

Antibody test	Result (AU)	Interpretation	Reference range	Sensitivity/specificity
Anti‐Scl‐70	0	Negative	≤ 5 AU, negative; > 5 AU, positive	65%/99%
Anti‐CENP A	1	Negative	≤ 5 AU, negative; > 5 AU, positive	11%/99%
Anti‐CENP B	1	Negative	≤ 5 AU, negative; > 5 AU, positive	13%/99%
Anti‐RP11	2	Negative	≤ 5 AU, negative; > 5 AU, positive	5%/100%
Anti‐RP155	1	Negative	≤ 5 AU, negative; > 5 AU, positive	7%/100%
Anti‐Fibrillarin	0	Negative	≤ 5 AU, negative; > 5 AU, positive	2%/100%
Anti‐NOR‐90	1	Negative	≤ 5 AU, negative; > 5 AU, positive ≤ 5 AU, negative; > 5 AU, positive	4%/99%
Anti‐Th/To	3	Negative	≤ 5 AU, negative; > 5 AU, positive ≤ 5 AU, negative; > 5 AU, positive	6%/98%
Anti‐PM‐Scl 100	0	Negative	≤ 5 AU, negative; > 5 AU, positive	7%/99%
Anti‐PM‐Scl 75	1	Negative	≤ 5 AU, negative; > 5 AU, positive	12%/98%
Anti‐Ku	3	Negative	≤ 5 AU, negative; > 5 AU, positive	6%/99%
Anti‐PDGFR	0	Negative	≤ 5 AU, negative; > 5 AU, positive	1%/100%
Anti‐Ro‐52	4	Negative	≤ 5 AU, negative; > 5 AU, positive	—

*Note:* Autoantibodies were assessed using an immunoblot assay (EUROLINE; EUROIMMUN AG, Lübeck, Germany). According to the laboratory reporting criteria, results of ≤ 5 AU were considered negative and > 5 AU positive. For grading of positive results, 6–10 AU was classified as borderline positive; 11–25 AU as positive (+); 26–50 AU as positive (++); and > 50 AU as strongly positive (+++). The sensitivity and specificity values shown are those reported by the laboratory for the respective antibody assays in relation to their association with systemic sclerosis. Scl‐70, DNA topoisomerase I; CENP, centromere protein; RP11, RNA polymerase III subunit RP11; RP155, RNA polymerase III subunit RP155; Th/To, Th/To ribonucleoprotein; PM‐Scl, polymyositis/scleroderma; PDGFR, platelet‐derived growth factor receptor; Ro‐52, 52‐kDa Ro/SSA antigen.

Abbreviation: AU, arbitrary units.

The clinical features relevant to the 2013 American College of Rheumatology/European League Against Rheumatism (ACR/EULAR) classification criteria for SSc were reviewed individually [6]. The sufficient criterion of skin thickening of the fingers extending proximal to the metacarpophalangeal joints was not met, as the patient had complete sparing of the hands and digits. Among the individual domains, there were no puffy fingers or sclerodactyly (0 points), no fingertip ulcers or pitting scars (0 points), no telangiectasias (0 points), and no SSc‐associated autoantibodies, including anticentromere, antitopoisomerase I (anti‐Scl‐70), or anti‐RNA polymerase III antibodies (0 points). Nailfold capillaroscopy was not performed, and pulmonary arterial hypertension or interstitial lung disease could not be evaluated because diffusing capacity of the lungs for carbon monoxide (DLCO) and high‐resolution computed tomography (HRCT) were not feasible for this patient; therefore, no points were assigned for these domains. Raynaud’s phenomenon was also absent (0 points). Thus, the patient did not meet the 2013 ACR/EULAR classification threshold for SSc.

Nailfold capillaroscopy was not performed during the acute admission and therefore could not be assessed. Noninvasive cutaneous imaging modalities, such as high‐frequency ultrasound (HFUS) or shear‐wave elastography, were also not performed during the acute admission or subsequent follow‐up visits. These investigations were not pursued because of cost constraints. Objective disease monitoring was instead conducted via serial clinical palpation using the modified Rodnan Skin Score (mRSS) alongside the baseline histopathology. Using the standard 17‐site mRSS criteria (0‐3 scale across 17 body areas; maximum score, 51), the patient’s baseline mRSS was 33/51 at presentation, demonstrating severe induration across the face, trunk, and bilateral extremities.

Systemic evaluation showed a serum creatinine level of 1.11 mg/dL and an unremarkable chest radiograph. However, in the absence of HRCT and diffusing capacity of the lung for carbon monoxide (DLCO), subclinical interstitial lung disease or other pulmonary involvement cannot be definitively excluded. Notably, cardiomegaly was observed, consistent with her known history of dilated cardiomyopathy. During her hospital stay, the patient also developed plantar foot ulcers. Lower‐limb arterial Doppler ultrasonography demonstrated mild atherosclerotic changes.

A skin biopsy was performed on the patient’s right leg, from an area of clinically indurated skin. An elliptical incisional skin biopsy measuring 0.6 × 0.5 × 0.3 cm was obtained from the right lower leg. Histopathological sectioning demonstrated tissue depth extending through the epidermis and full thickness dermis into the superficial subcutaneous fat (subcutis). The underlying deep muscular fascia and skeletal muscle were not included in the specimen. Haematoxylin and eosin (H&E) staining revealed thinning of the epidermis, superficial papillary dermal edema, thickening of the papillary and reticular dermis, with broad collagen bundles arranged horizontally and parallel to the overlying epidermis. Sparse lymphocytic infiltrates in the perivascular and periadnexal regions were also seen (Figure 2). Periodic acid–Schiff–Alcian blue (PAS‐AB) staining demonstrated abundant interstitial dermal mucin, with the Alcian blue component at pH 2.5 highlighting the mucin as blue‐green material within the dermis (Figure 3). Taken together, the clinical and histopathological findings supported a diagnosis of scleredema diabeticorum. The posterior neck, upper back, and shoulders were specifically examined and showed no evidence of cutaneous induration. Thus, although these are recognized sites of scleredema involvement, they were not affected in the present case, contributing to the atypical distribution of the disease.

**FIGURE 2:**
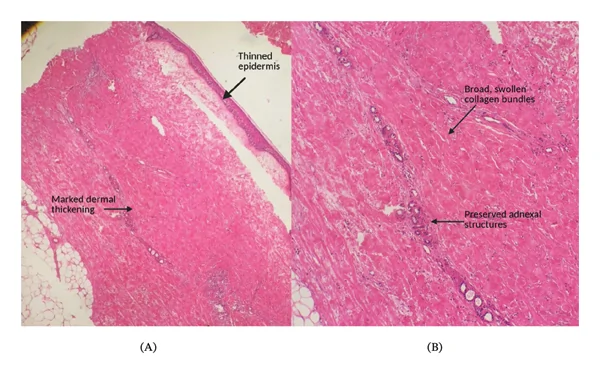
Histopathological findings are consistent with scleredema diabeticorum. (A) Thinned epidermis overlying a markedly thickened dermis with broad, swollen collagen bundles (haematoxylin and eosin [H&E], magnification: × 100). (B) Broad, swollen collagen bundles extending into the deep dermis with preservation of adnexal structures (H&E, magnification: × 400).

**FIGURE 3:**
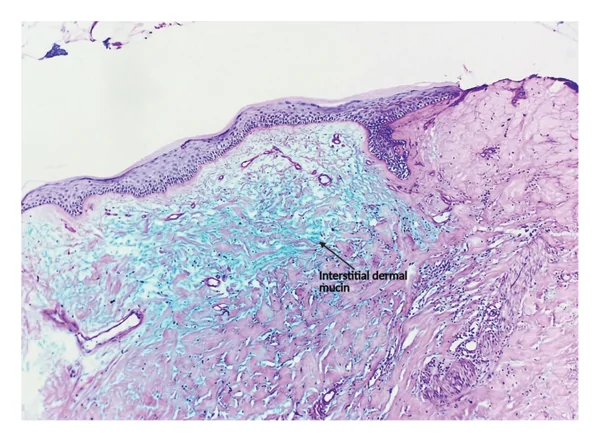
Periodic acid–Schiff–Alcian blue (PAS‐AB) staining demonstrating abundant interstitial dermal mucin. Alcian blue at pH 2.5 highlights the mucin as blue‐green material separating the thickened collagen bundles (magnification: × 200).

Further investigations were performed to evaluate alternative causes of generalized skin induration. Serum protein electrophoresis and immunofixation showed no detectable monoclonal protein, while serum free kappa and lambda light chains and the kappa/lambda ratio were within normal limits (Tables 3 and 4). The absolute eosinophil count was 0 × 10^9^/L (0% of leukocytes), and no groove sign was present, making eosinophilic fasciitis less likely; however, it could not be definitively excluded because the biopsy did not include the deep fascia and no fascial imaging was performed. Thyroid function testing was also normal, with a thyroid‐stimulating hormone (TSH) level of 0.51 µIU/L and normal free thyroxine (T4) of 1.2 ng/dL, arguing against hypothyroidism as a cause of the generalized nonpitting edema and skin changes. Venous Doppler ultrasonography of the lower limbs showed compressible veins with appropriate augmentation on distal compression and adequate venous filling, with no evidence of thrombus or collateral circulation, making venous stasis‐related disease less likely to be the primary explanation for the generalized skin induration. A detailed medication history was reviewed to assess for drug‐induced pseudoscleroderma. The patient had no history of exposure to medications known to cause scleroderma‐like cutaneous changes, including bleomycin, taxanes (paclitaxel or docetaxel), gemcitabine, pentazocine, warfarin or other vitamin K antagonists, interferon‐α, or bromocriptine.

**TABLE 3:** Serum protein electrophoresis showed no detectable paraprotein.

Investigation	Patient result	Reference range	Interpretation
Total protein	6.9 g/dL	6.6–8.7 g/dL	Normal
Albumin	3.9 g/dL	3.2–5.5 g/dL	Normal
Alpha‐1 globulin	0.3 g/dL	0.1–0.4 g/dL	Normal
Alpha‐2 globulin	1.1 g/dL	0.4–1.2 g/dL	Normal
Beta globulin	0.8 g/dL	0.5–1.1 g/dL	Normal
Gamma globulin	0.9 g/dL	0.5–1.6 g/dL	Normal
A/G ratio	1.2	1.2–1.7	Normal

*Note:* Total protein, albumin, alpha‐1 globulin, alpha‐2 globulin, beta globulin, gamma globulin, and the albumin‐to‐globulin (A/G) ratio were within the respective laboratory reference ranges.

**TABLE 4:** Serum free light‐chain analysis showing normal free kappa, free lambda, and kappa/lambda ratio.

Test	Result	Reference range	Interpretation
Free Kappa	17.36 mg/L	3.3–19.4 mg/L	Normal
Free Lambda	14.66 mg/L	5.71–26.3 mg/L	Normal
Kappa/Lambda ratio	1.18	0.26–1.65	Normal

The initial lower‐limb edema occurred in the setting of hypoalbuminemia and diabetic nephropathy and improved with diuresis, whereas the subsequent persistent skin induration, progressive proximal extension, and histopathological findings supported scleredema diabeticorum rather than edema alone. Erosions, crusting, and yellow discharge were treated as superimposed bacterial infection and did not account for the underlying diffuse skin induration. Mild peripheral arterial disease identified on arterial Doppler was considered insufficient to explain the generalized cutaneous changes, while venous Doppler showed no evidence of venous thrombosis. Stasis‐related changes or lipodermatosclerosis were therefore considered less likely as the primary explanation. Nephrogenic systemic fibrosis was also considered less likely in the absence of gadolinium exposure and characteristic histopathological findings, although it could not be definitively excluded. The additional clinical, laboratory, imaging, and histopathological findings were considered in evaluating nephrogenic systemic fibrosis, eosinophilic fasciitis, Type 2 scleredema, scleromyxedema, hypothyroidism, morphea, lipodermatosclerosis/stasis dermatitis, and drug‐induced pseudoscleroderma as summarized in Table 5.

**TABLE 5:** Differential diagnoses considered in the present case and the clinical, laboratory, imaging, and histopathological findings supporting or arguing against each diagnosis.

Differential diagnosis	Findings in the present case	Assessment
Nephrogenic systemic fibrosis	No history of gadolinium‐based contrast exposure; biopsy showed no characteristic spindle‐cell proliferation	Considered less likely
Eosinophilic fasciitis	Normal absolute eosinophil count; no groove sign; hands and feet spared; deep fascia not sampled	Less likely; fascial involvement could not be definitively assessed histologically
Type 2 scleredema/scleromyxedema	SPEP and immunofixation negative for monoclonal protein; normal free kappa, free lambda and *κ*/*λ* ratio; no characteristic papular lesions	Monoclonal gammopathy not identified; scleromyxedema not supported clinically
Hypothyroidism/myxedema	Normal TSH (0.51 µIU/L) and free T4 (1.2 ng/dL)	Not supported by normal thyroid function tests
Morphea/Deep morphea	Diffuse and symmetric rather than localized plaque‐like induration; histopathology not supportive	Less likely
Lipodermatosclerosis/Stasis dermatitis	Lower‐limb involvement present; venous Doppler showed compressible veins, appropriate augmentation on distal compression, adequate filling, and no thrombus or collateral circulation	Less likely as the primary explanation for generalized induration
Drug‐induced pseudoscleroderma	No history of exposure to medications recognized to cause scleroderma‐like cutaneous changes, including bleomycin, taxanes (paclitaxel or docetaxel), gemcitabine, pentazocine, warfarin or other vitamin K antagonists, interferon‐α, or bromocriptine	Less likely; no apparent pharmacologic trigger identified

Following review of her clinical history and biopsy results, the diagnosis was revised to scleredema diabeticorum. Initial inpatient management in May 2025 consisted of a short, 11‐day course of oral cyclosporine (100 mg twice daily; approximately 2.5–3.0 mg/kg/day) together with prednisolone taper (from 15 mg to 10 mg daily) to control severe acute edema, induration, and pain. Cyclosporine was selected as short‐term anti‐inflammatory therapy rather than long‐term maintenance because rapid control of the severe inflammatory presentation was required, while prolonged immunosuppression was considered less desirable in the setting of concurrent acute non‐ST‐elevation myocardial infarction (NSTEMI), reduced left ventricular ejection fraction (30%), chronic kidney disease, and diabetic nephropathy (urine albumin‐to‐creatinine ratio of 248 mg/g). Concurrent cardiac therapy included sacubitril/valsartan (25 mg twice daily), carvedilol (3.125 mg twice daily), and furosemide (40 mg daily).

Safety monitoring during cyclosporine therapy included serial assessment of renal function, blood pressure, serum electrolytes, and cyclosporine trough concentrations. Blood pressure remained controlled throughout the treatment period, with no documented hypertensive complications. Serum creatinine remained within the reference range without an interval increase, measuring 1.11 mg/dL at baseline, 0.79 mg/dL during therapy, and 0.75 mg/dL after discharge (reference range, 0.57–1.11 mg/dL), with no evidence of acute nephrotoxicity during the treatment period. Serum potassium ranged from 3.4 to 3.7 mEq/L, while magnesium remained stable at 2.12 mg/dL. Cyclosporine trough concentration (C0) was 94.7 ng/mL on 26 May 2025 and 149 ng/mL on 4 June 2025, both within the laboratory reference range of 50–350 ng/mL. As an indicator of inflammatory activity, high‐sensitivity C‐reactive protein decreased from an admission peak of 6.28 mg/L to 2.15 mg/L following treatment (reference, < 5.0 mg/L).

UVA1 phototherapy was attempted early during admission, but the patient developed immediate intolerance characterized by severe heat discomfort, marked facial flushing, profuse diaphoresis, and light‐headedness within minutes of initiating exposure. The session was immediately aborted because of thermal distress, and her symptoms resolved completely within 1 hour after cessation of exposure, active cooling with cool compresses, and oral hydration. As treatment was aborted at the initial exposure, a complete UVA1 treatment course was not established, and the device‐specific wavelength, dose per session, escalation protocol, number and frequency of sessions, and cumulative dose were therefore not available for reporting. Accordingly, the clinical course cannot be interpreted as a failure of an adequately dosed UVA1 regimen. Intravenous immunoglobulin was not administered because of the patient’s acute cardiac status and concern regarding volume‐related complications. Although cyclosporine and UVA1 were used during the same inpatient period, UVA1 exposure was limited to the initial attempted treatment because of intolerance, precluding prolonged concurrent phototherapy and immunosuppression.

Following discharge, the patient was transitioned to maintenance therapy with subcutaneous methotrexate (15 mg weekly), folic acid (5 mg daily), colchicine (0.5 mg twice daily), and a structured tapering course of oral prednisolone, starting at 10 mg daily. Prednisolone was prescribed as a short‐term anti‐inflammatory bridge, given the severity and functional impact of the cutaneous disease, her inability to tolerate UVA1 phototherapy, and the delayed clinical response generally expected with methotrexate therapy. Prednisolone was maintained at 10 mg daily for 4 weeks, followed by reductions of 2.5 mg every 2–4 weeks, with the taper completed over 12–16 weeks. In view of the anticipated glucocorticoid‐related effect on glycemic control, insulin therapy was intensified to a basal‐bolus regimen with insulin glargine (Lantus, 22 units nightly) and regular insulin (Humulin‐R, 12 units three times daily). Glycemic control remained stable without acute metabolic decompensation.

The patient was followed for 10 months after discharge, from May 2025 to March 2026, and demonstrated sustained clinical improvement in her cutaneous manifestations. She remained clinically stable on subcutaneous methotrexate (15 mg weekly), folic acid (5 mg daily), and colchicine (0.5 mg twice daily). Her baseline mRSS of 33/51 decreased to 18/51 on an interim assessment in November 2025, accompanied by clinically evident softening of the previously indurated skin and visible improvement on paired clinical photographs. Serial mRSS assessments were used as a pragmatic clinical measure to document changes in skin thickness over the course of treatment. However, mRSS has not been specifically validated as an outcome measure for scleredema diabeticorum.

During the same period, she developed a diabetic foot ulcer in the setting of pre‐existing peripheral arterial disease, complicated by osteomyelitis and tissue necrosis, ultimately requiring a right below‐knee amputation in November 2025. The amputation subsequently limited further formal mRSS assessment of the affected lower limb. At the 10‐month follow‐up, the patient remained clinically stable with continued improvement of the remaining cutaneous manifestations. Renal function remained preserved, with a serum creatinine of 0.34 mg/dL at 10 months. Blood pressure remained well controlled, averaging 130/90 mmHg. Glycemic control also remained stable, with a follow‐up HbA1c of 7.5% compared with 7.8% at baseline. Table 6 shows the CARE timeline of this patient’s clinical problem.

**TABLE 6:** CARE timeline of the patient’s clinical course.

Date/time	Clinical events
May 2024	Cerebrovascular accident (CVA) resulting in transient diplopia, which resolved spontaneously.
November 2024	Developed generalized body swelling, including periorbital puffiness and bilateral lower‐limb edema. Evaluation demonstrated hypoalbuminemia and elevated serum creatinine, consistent with diabetic nephropathy. Furosemide was initiated, with subsequent improvement in edema.
Following resolution of edema	Developed skin lesions and progressive induration of the lower extremities, which gradually extended upward to the infraumbilical region and subsequently involved the upper limbs and face. The affected areas became increasingly stiff and were associated with pruritus, scaling, erosions/crusting, and intermittent yellowish discharge. Secondary bacterial infection was treated with oral antibiotics and topical therapy.
Early May 2025	Progressive, symmetric cutaneous induration prompted rheumatology referral. Systemic sclerosis was initially suspected following a positive ANA, and a tapering regimen of deflazacort was initiated. The patient remained on corticosteroid therapy at the time of hospital presentation.
19 May 2025	Hospital admission with NSTEMI, dilated cardiomyopathy (LVEF 30%), severe “woody” skin induration, tissue edema, and acute plantar foot ulceration.
20–22 May 2025	Diagnostic evaluation for systemic sclerosis and alternative causes of generalized skin induration. SSc‐associated autoantibodies were negative apart from low‐positive ANA and positive antismooth muscle antibody; paraproteinemia and hypothyroidism were not identified. Absolute eosinophil count was normal, and no groove sign was present.
23 May 2025	Scalpel incisional biopsy of the right lower leg demonstrated epidermal thinning, dermal thickening, broad/horizontally arranged collagen bundles, and sparse lymphocytic infiltrates, suggesting scleredema diabeticorum.
24 May−4 June 2025	Treated with an 11‐day course of cyclosporine. Renal function, blood pressure, electrolytes, and cyclosporine trough concentrations were monitored. Glycemic management was intensified.
05 June 2025	Hospital discharge on maintenance methotrexate, folic acid, colchicine, and intensified insulin therapy along with prednisolone. Baseline mRSS was 33/51.
November 2025	Six‐month follow‐up: mRSS improved to 18/51, with clinical softening of the previously indurated skin. During this period, the patient developed diabetic foot ulceration complicated by osteomyelitis and tissue necrosis, ultimately requiring a right below‐knee amputation.
March 2026	Ten‐month follow‐up: sustained clinical improvement of the remaining cutaneous induration while continuing methotrexate, folic acid and colchicine. Renal function and glycemic control remained stable, with serum creatinine 0.34 mg/dL and HbA1c 7.5%.

## 3. Discussion

Scleredema diabeticorum is an uncommon but well‐recognized complication of long‐standing diabetes, characteristically manifesting as symmetric, “woody” induration of the skin with a predilection for the posterior neck, shoulders, and upper back. Unlike SSc, which frequently involves the distal extremities, scleredema typically spares the hands and feet [7]. Our patient’s presentation differed from the classic pattern, as she was a 75‐year‐old woman, whereas scleredema diabeticorum predominantly affects middle‐aged men, with a reported male‐to‐female ratio of approximately 10:1, and her initial involvement was noted in the limbs and face rather than the upper trunk. Nevertheless, her 20‐year history of Type 2 diabetes mellitus requiring insulin therapy, complicated by diabetic nephropathy, together with a prior cerebrovascular accident, aligns with the profile of Type 3 scleredema (diabeticorum) in which chronic hyperglycemia promotes nonenzymatic glycosylation of collagen and contributes to progressive dermal thickening and fibrosis [1, 2, 4].

The clinical overlap between scleredema and SSc presents a notable diagnostic challenge, particularly when skin tightness and reduced joint mobility are prominent. SSc commonly features sclerodactyly, Raynaud’s phenomenon, digital ulcers, and visceral involvement such as pulmonary fibrosis or scleroderma renal crisis; these features were absent in our patient [8].

Her serologic workup revealed only a nonspecific positive ANA and antismooth muscle antibody, with negative SSc‐associated autoantibodies, underscoring that ANA positivity alone is neither diagnostic of SSc nor specific to autoimmune disease. Although the absence of SSc‐associated autoantibodies reduced support for SSc, it did not, in isolation, exclude the diagnosis [9]. In this context, the 2013 ACR/EULAR criteria are intended primarily for classification and research purposes and should not be used in isolation to establish or exclude a clinical diagnosis [6].

Although HFUS and shear‐wave elastography have been proposed as noninvasive approaches for quantifying skin thickness and stiffness in sclerosing disorders, their validity remains incompletely established. In a recent study of patients with SSc, Desai et al. found that HFUS‐based measurements showed only limited correlations with the modified Rodnan skin score and histopathological parameters; although ultrasound‐derived dermal thickness demonstrated a moderate correlation with histologic dermal thickness, it did not correlate significantly with collagen density or fibrosis‐related gene expression. These findings suggest that HFUS cannot yet be considered a consistently validated surrogate for clinical or histopathological assessment of cutaneous fibrosis. Accordingly, in the present case, the initial diagnosis was supported by histopathological examination, while serial clinical assessment using the mRSS was used to document disease severity and treatment response [10].

Histopathology provides important supportive evidence in distinguishing these entities. In scleredema diabeticorum, biopsy characteristically shows a thinned epidermis overlying a markedly thickened dermis with broad, swollen collagen bundles separated by mucin‐containing spaces; inflammatory infiltrates are typically sparse and perivascular [11–13]. By contrast, SSc skin exhibits early dermal edema with perivascular CD4^+^ T‐lymphocyte infiltrates, bland intimal proliferation of small vessels, capillary rarefaction, and later pronounced perivascular and interstitial fibrosis from compact collagen and hyaluronic acid accompanied by loss of dermal capillaries, skin appendages, lymphatics, and subcutaneous fat [1]. In our patient, H&E staining demonstrated superficial edema, broad thickened collagen bundles, and scant perivascular lymphocytes, while PAS–Alcian blue staining demonstrated abundant interstitial dermal mucin. These findings provided additional histopathological support for a clinicopathological diagnosis of scleredema diabeticorum. Apart from the cutaneous manifestations, the patient had dilated cardiomyopathy with a left ventricular ejection fraction of 30%. Although her long‐standing diabetes mellitus, hypertension, peripheral atherosclerosis, and NSTEMI provide more likely explanations for the cardiomyopathy, similar cardiac involvement has been reported in patients with scleredema. Therefore, the cardiomyopathy is presented as a coexisting clinical finding rather than being attributed causally to scleredema [14].

Treatment of scleredema diabeticorum remains largely empirical, with evidence derived predominantly from case reports and small case series. Phototherapy, particularly PUVA and UVA1, has shown favorable responses in reported cases, with clinical improvement reported in 12 of 14 patients treated with PUVA and 13 of 14 treated with UVA1 in a recent scoping review. Reported UVA1 regimens typically involved 30–60 J/cm^2^ per session, administered three to five times weekly for 12‐24 sessions, with cumulative doses of approximately 1200–1800 J/cm^2^ [13]. However, these findings should be interpreted cautiously given the limited and heterogeneous evidence base. In our patient, UVA1 was attempted early but was discontinued after immediate intolerance and therefore could not be adequately assessed. Intravenous immunoglobulin has also been associated with improvement in a small number of reported cases but was not administered in our patient because of concern regarding volume‐related complications in the setting of acute NSTEMI, reduced LVEF (30%), and diabetic nephropathy. Methotrexate has demonstrated variable efficacy, with improvement reported in eight of 22 patients, although responses were more frequently described when methotrexate was used in combination with other therapies rather than as monotherapy [13]. Colchicine has also been used as an adjunctive therapy, with proposed antifibrotic effects related to interference with collagen transport and secretion and suppression of TGF‐β‐induced Type I collagen expression [15]. Given the severity of this patient’s disease and the inability to tolerate UVA1, systemic therapy was considered appropriate. In a multicenter study of 44 patients with scleredema, systemic corticosteroids and immunosuppressive therapies were similarly reserved for patients with severe disease in whom phototherapy had failed [16]. A short inpatient course of cyclosporine was therefore used for acute disease control, followed by maintenance therapy with methotrexate, colchicine, and low‐dose prednisolone. Although continued corticosteroid therapy posed a potential risk of worsening glycemic control, this was anticipated and managed through intensification of insulin therapy and close metabolic monitoring. The patient subsequently demonstrated sustained clinical improvement, with the mRSS decreasing from 33/51 to 18/51 at 6 months. Because multiple therapies were administered sequentially and concurrently, the improvement cannot be attributed to any single agent.

The principal contribution of this case lies in the combination of several atypical clinical and therapeutic features. The patient was a 75‐year‐old woman, whereas the diabetes‐associated form of scleredema is more commonly reported in middle‐aged men. In addition, the disease initially manifested in the lower extremities and progressed in an ascending pattern rather than beginning in the posterior neck, shoulders, or upper back. Prominent facial and periorbital involvement further broadened the clinical presentation, while the hands and feet remained spared. The degree of cutaneous involvement occurred despite a relatively modest HbA1c of 7.8%, illustrating that extensive scleredematous disease may occur even when current glycemic indices do not reflect severe hyperglycemia. Finally, management was complicated by diabetic nephropathy and a concurrent NSTEMI with reduced left ventricular ejection fraction, substantially limiting the use of systemic therapies and necessitating individualized short‐term immunomodulatory treatment and close safety monitoring. Thus, the case adds to the clinical spectrum of scleredema diabeticorum by illustrating an atypical distribution and demographic profile together with substantial cardiovascular and renal constraints on treatment.

This case has several limitations. The absence of nailfold capillaroscopy limited assessment for microvascular changes associated with SSc, while the lack of HFUS and shear‐wave elastography limited objective longitudinal assessment of skin thickness and tissue stiffness. HRCT and pulmonary function testing with DLCO were not performed to assess for subclinical SSc‐related pulmonary involvement. The skin biopsy extended through the dermis into the superficial subcutis but did not include the deep fascia, limiting assessment of deeper fascial involvement. Finally, formal mRSS assessment became less comprehensive after right below‐knee amputation because the affected lower‐limb sites were no longer fully available for longitudinal scoring.

## Author Contributions

Muhammad Waleed Imran contributed to conceptualization, data curation, literature review, writing of the original draft, review and editing of the manuscript, and project administration. Sanwal Sardar Nawaz contributed to conceptualization, data curation, literature review, writing of the original draft, and review and editing of the manuscript. Hina Abbasi contributed to clinical investigation, clinical data curation, clinical interpretation, writing of the original draft, and review and editing of the manuscript. Farah Khurshid, Waqar Khattak, and Tahir Iqbal contributed to clinical supervision, clinical interpretation, and review and editing of the manuscript.

## Funding

The authors received no specific funding for this work.

## Ethics Statement

This case report was reviewed and approved by the Institutional Review Board (IRB) of Shifa International Hospital, Islamabad, Pakistan (IRB 403‐25).

## Consent

This case report was prepared in accordance with the CARE guidelines for case reports. Written informed consent was obtained from the patient for publication of the case report, accompanying clinical information, and clinical photographs.

## Conflicts of Interest

The authors declare no conflicts of interest.

## Patient Perspective

When my skin first began to feel hard and tight, I found it very distressing. The increasing stiffness made everyday movements and even simple tasks such as taking care of myself difficult and painful. It was difficult to understand and accept such a sudden and unusual change, especially after living with diabetes for many years. Receiving a clear diagnosis brought me relief because I finally understood what was happening to my skin. The treatment was not easy, and my recovery was further complicated by the need for a leg amputation because of my underlying circulation problems. Thankfully, treatment significantly improved the tightness, pain, and swelling, and my skin has remained much softer and more comfortable. Although I am currently less mobile and unable to do many of the things I used to do independently, I am grateful for the improvement in my skin and hopeful that continued rehabilitation will help me regain some of my independence.

## Data Availability

All data relevant to this case report are included in the published article. Additional de‐identified clinical information may be obtained from the corresponding author upon reasonable request, subject to patient confidentiality and institutional policies.
